# PESTO: Parameter EStimation TOolbox

**DOI:** 10.1093/bioinformatics/btx676

**Published:** 2017-10-23

**Authors:** Paul Stapor, Daniel Weindl, Benjamin Ballnus, Sabine Hug, Carolin Loos, Anna Fiedler, Sabrina Krause, Sabrina Hroß, Fabian Fröhlich, Jan Hasenauer

**Affiliations:** 1 Institute of Computational Biology, Helmholtz Zentrum München, Neuherberg, Germany; 2 Center for Mathematics, Technische Universität München, Garching, Germany

## Abstract

**Summary:**

PESTO is a widely applicable and highly customizable toolbox for parameter estimation in MathWorks MATLAB. It offers scalable algorithms for optimization, uncertainty and identifiability analysis, which work in a very generic manner, treating the objective function as a black box. Hence, PESTO can be used for any parameter estimation problem, for which the user can provide a deterministic objective function in MATLAB.

**Availability and implementation:**

PESTO is a MATLAB toolbox, freely available under the BSD license. The source code, along with extensive documentation and example code, can be downloaded from https://github.com/ICB-DCM/PESTO/.

**Supplementary information:**

Supplementary data are available at *Bioinformatics* online.

## 1 Background

In many disciplines such as computational biology, engineering and operations research, researchers are faced with the problem of finding a set of parameters optimizing a given objective function and determining uncertainties of those estimates. There are various toolboxes available which offer this functionality, however, most of them focus either on individual aspects or are tailored to specific model types, e.g. ordinary differential equations (ODEs) or partial differential equations (PDEs). Hence, the user must adapt to different tools, depending on the current problem structure. A feature comparison of a selection of toolboxes related to parameter estimation is provided as Supplementary Table S1. In an effort to provide a universally applicable parameter estimation toolbox, we developed PESTO. PESTO is a freely available MATLAB toolbox offering scalable state-of-the-art algorithms for optimization, uncertainty and identifiability analysis, which do not depend on any problem-specific assumptions.

## 2 Features

PESTO offers the following features (Supplementary Fig. S1):

- Multi-start local optimization and interfaces to global and hybrid optimizers
- Optimization-based, integration-based or hybrid profile calculation for uncertainty and identifiability analysis
- Several sampling methods for uncertainty and identifiability analysis
- Visualization of all analysis results
- Efficient work flow and optional parallelization
- High customizability

PESTO is agnostic of the underlying model or objective function and can be used in conjunction with any deterministic optimization problem formulated as a MATLAB function. The objective function is treated as black box, no further assumptions are made. The optimization can be accelerated when gradient and Hessian of the objective function are provided. PESTO can be applied to many optimization problems within the systems biology field and beyond.

### 2.1 Optimization

Many biological problems are non-convex and exhibit several local optima of which usually the global one is of interest. These optima cannot be derived analytically and evaluating the whole parameter space is computationally prohibitive. Therefore, there are diverse stochastic, deterministic or hybrid algorithms available (Raue *et al.*, 2013). To deal with multiple local optima, PESTO provides a multi-start local optimization strategy which has been shown to perform well in terms of convergence and computation time for biological ODE and PDE models (Hross and Hasenauer, 2016; Raue *et al.*, 2013). The user can choose between different strategies for automated starting point selection, such as latin hypercube or uniform sampling, or provide custom starting points. Multi-start local optimization usually performs very well in terms of finding the assumingly global optimum (Raue *et al.*, 2013) (Supplementary Fig. S2). Nevertheless, PESTO additionally provides access to two global optimization toolboxes, MEIGO and PSwarm (Egea *et al.*, 2014; Vaz and Vicente, 2007) through a uniform interface.

### 2.2 Identifiability and uncertainty analysis

Optimization problems arising in biology are often ill-posed and the estimated parameters are subject to large uncertainties. PESTO provides profile calculation and Markov-chain Monte Carlo methods to assess the properties of the objective function and the uncertainty and practical identifiability of parameters as well as any functions thereof. For profile calculation, the user can choose between optimization- and integration-based or hybrid profile calculation methods which provide maximum projections of the objective function (Boiger *et al.*, 2016). The novel hybrid profile calculation method provides the robustness of optimization-based profile calculation and the efficiency of integration-based profile calculation for which, to the best of our knowledge, no other implementation exists so far. Markov-chain Monte Carlo methods sample from parameter distributions, e.g. posteriors arising in Bayesian inference. PESTO supports the classical Metropolis-Hastings algorithm, two adaptive Metropolis-Hastings algorithms, the Metropolis-adjusted Langevin algorithm as well as the schemes from the MATLAB-based Delayed Rejection Adaptive Metropolis (DRAM) toolbox (Haario *et al.*, 2006). Additionally, PESTO provides multi-chain algorithms parallel tempering (Lacki and Miasojedow, 2015; Vousden *et al.*, 2016) and parallel hierarchical sampling (Rigat and Mira, 2012). In case profile calculation and sampling are computationally too expensive, PESTO can perform uncertainty analysis based on local quadratic approximations which are, however, less reliable. PESTO is the first toolbox to provide generic implementations of several of the above-mentioned algorithms for MATLAB or in general (Supplementary Table S1).

### 2.3 Visualization

For all above-mentioned analyses, PESTO generates various kinds of result plots: line plots, parallel coordinate plots, scatter plots and bar plots, some of which are shown in Supplementary Figure S1. Plots for optimization, parameter sampling and profile calculation are updated continuously to monitor progress of the computations. All plots are extensively customizable and can be exported as vector or raster graphics.

## 3 Examples, documentation and scalability

PESTO comes with extensive documentation and examples demonstrating all of its functions. Examples include several ODE and PDE models from recent research in computational biology, such as Fröhlich *et al.* (2017) or Boiger *et al.* (2016). The dimensionality of the example problems included in PESTO ranks among the highest currently found in systems biology and demonstrates the scalability of the implemented methods (Supplementary Fig. S3).

PESTO has been successfully applied in more than 10 published studies referenced on the project website. Although these case studies come from computational biology, PESTO is also applicable in other research fields.

Due to the high abstraction level of PESTO, it does not directly support systems biology model definition standards such as the systems biology markup language (SBML). However, such models can easily be analyzed using PESTO in combination with AMICI, a toolbox for scalable simulation of ODE systems (e.g. Fröhlich *et al.*, 2017). AMICI handles the time discretization necessary for ODEs and allows also for the simulation of discretized PDEs (Serban and Hindmarsh, 2005), which is demonstrated in the examples included in PESTO.

## 4 Implementation and availability

PESTO is implemented fully in MATLAB and compatible with recent MATLAB releases running on Windows, Mac and Linux. For local optimization, PESTO requires the MATLAB ‘Optimization Toolbox’ (MathWorks). With the MATLAB ‘Parallel Computing Toolbox’ or ‘Distributed Computing Toolbox’ (MathWorks) installed, several PESTO routines can make use of multi-core processors.

PESTO is released under a BSD license. The source code, license terms, extensive documentation and example code are available on https://github.com/ICB-DCM/PESTO/.

## 5 Conclusion

Here we presented PESTO, a toolbox for many aspects of parameter estimation in MATLAB. Its strengths are the combination of many state-of-the-art methods which are applicable to a wide array of different parameter estimation problems through a single interface. This facilitates a synergistic use and an in-depth analysis of problems (Hug *et al.*, 2013).

## Funding

This project has received funding through the European Union‘s Horizon 2020 research and innovation programme under grant agreement no. 686282, the German Federal Ministry of Education and Research under the grant agreement no. 01ZX1310B and the German Research Foundation via the Graduate School of Quantitative Biosciences Munich (QBM).

*Conflict of Interest*: none declared.

## Supplementary Material

Supplementary Data
