# Supplementary material for: PESTO: Parameter EStimation TOolbox
Source: Bioinformatics. 2017 Oct 23;34(4):705–7. doi: 10.1093/bioinformatics/btx676 (PMC5860618; doi:10.1093/bioinformatics/btx676)
Supplement: Supplementary Data [file btx676_supp.zip › btx676-suppl_data/PESTO_Supplement.pdf]

Supplementary figures to
PESTO: Parameter EStimation TOolbox

Paul Stapor, Daniel Weindl, Benjamin Ballnus, Sabine Hug, Carolin Loos, Anna Fiedler, Sabrina Krause, Sabrina Hroß, Fabian Fröhlich, and Jan Hasenauer

List of Figures

1 PESTO features and workflow overview with visualization examples. 2
2 Comparison of three different optimizers 3
3 Comparison of the size of example models included in PESTO with BioModels 4

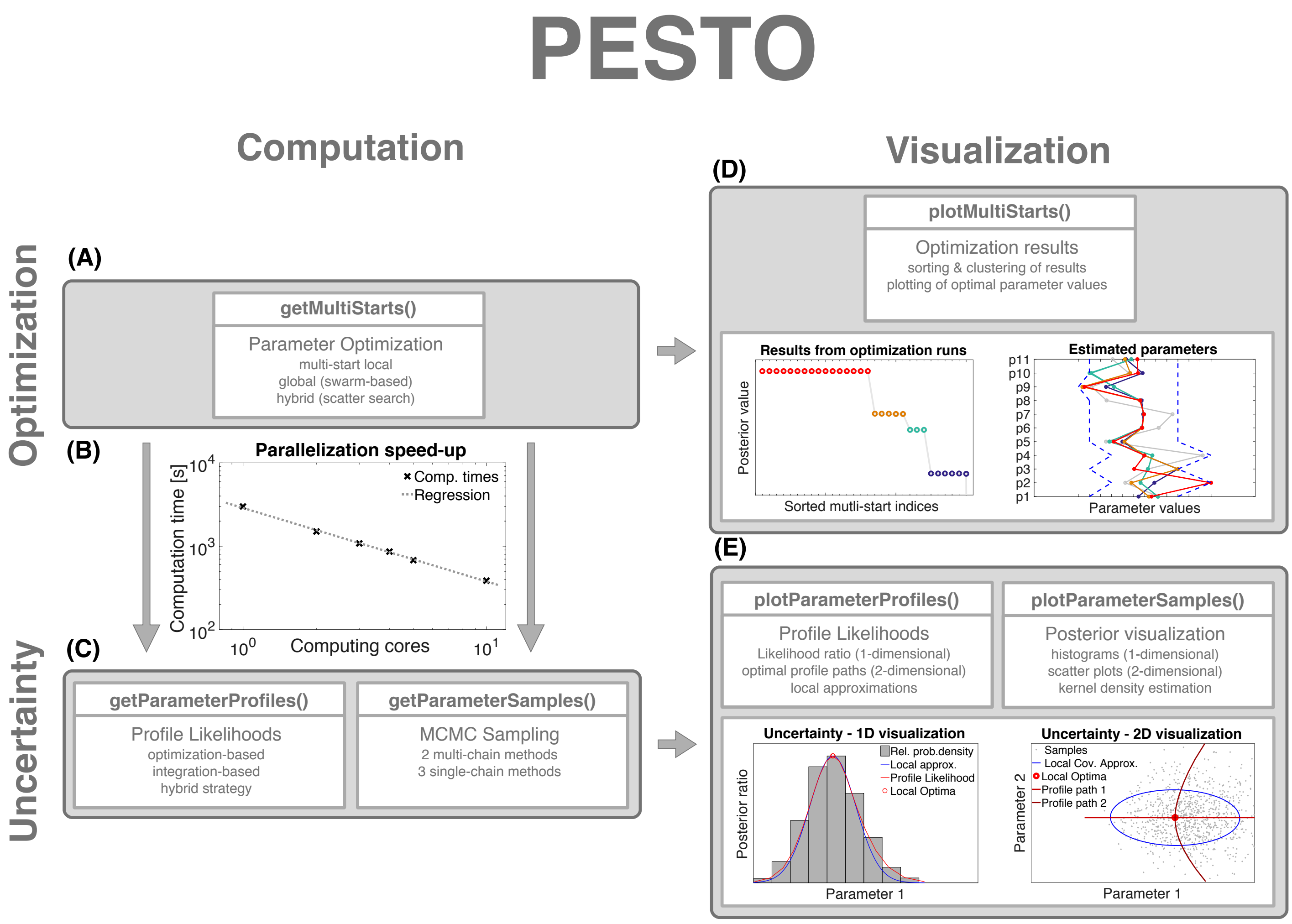

Supplementary Figure 1: PESTO features and workflow overview with visualization examples.
(A) In a parameter estimation problem, usually the best parameter value is found by solving an optimization problem. In PESTO, this is done by calling the routine `getMultiStarts()`, which is able to perform parameter optimization by using either a global, a multi-start local optimization or a hybrid optimization technique.
(B) Optimization and uncertainty analysis can be accelerated by using parallelization, if the MATLAB parallel computing toolbox is installed. The figure shows the speed-up for the PESTO example problem `jakstat_signaling` on an fmincon optimization with 25 multi-starts, using 1 to 10 CPU cores.
(C) Uncertainty analysis in PESTO can be carried out by the routines `getParameterProfiles()` or `getParameterSamples()`. `getParameterProfiles()` computes profile likelihoods, starting from the best found optimum using either a classical optimization-based, a more recent integration-based or a completely new hybrid approach for profile computation. `getParameterSamples()` samples parameter values based on Markov-Chain Monte-Carlo methods. Here, the user has the choice between two multi-chain methods (parallel tempering or parallel hierarchical sampling) and three single chain methods (adaptive Metropolis(-Hastings), delayed rejection adaptive Metropolis, Metropolis-adjusted Langevin algorithm). The delayed rejection adaptive Metropolis sampling algorithm needs the MATLAB toolbox DRAM to be installed.
(D) Optimization results can be visualized using the routine `plotMultiStarts()`. By default, it plots the sorted objective values from the optimization runs (which gives the user a tool to judge the quality of the optimization convergence) and the parameter values, sorted vertically, with their bounds for each optimization run.
(E) Results from uncertainty analysis can be visualized by either `plotParameterProfiles()` or `plotParameterSamples()`. Both routines can be used to assess parameter uncertainties in either one parameter or in two parameters jointly. Profiles and local approximations can be visualized in 1D as posterior ratio or a histogram drawn from the samples visualizes the relative marginal probability density. In two dimensions, profile paths can be plotted, samples as either a scatter plot or a kernel density estimate and local approximations of the covariance structure can be shown.

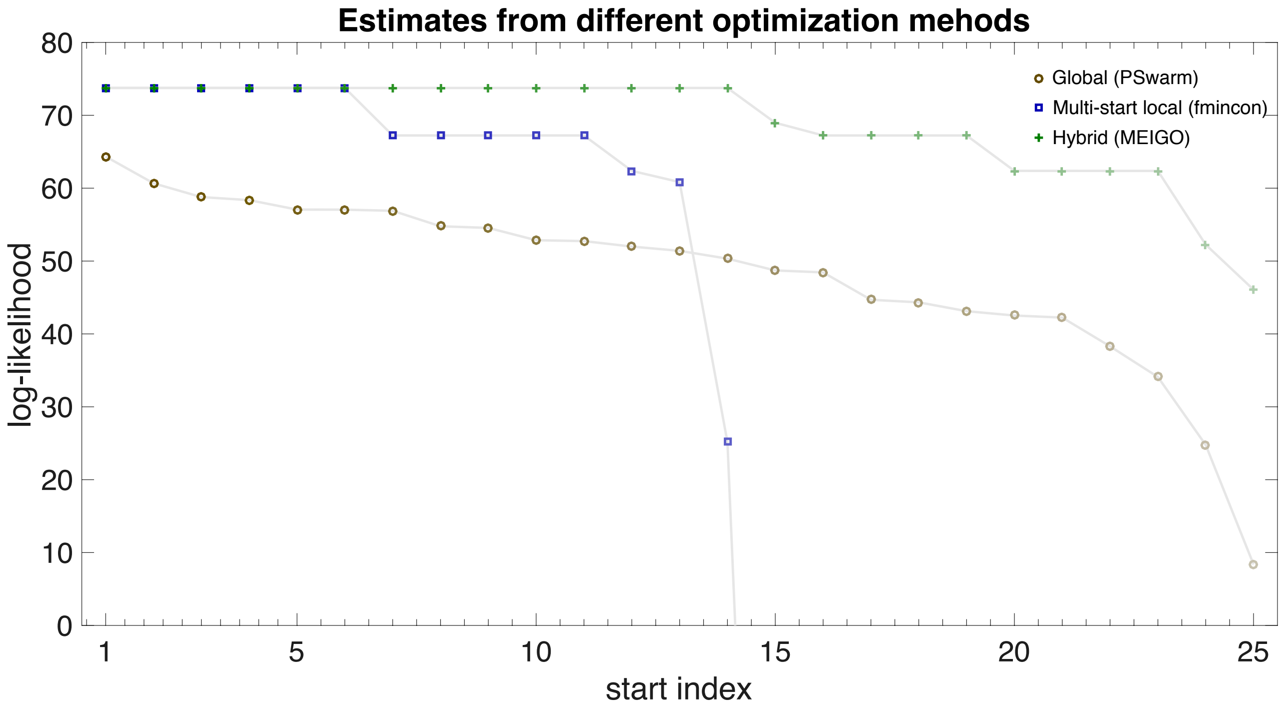

Supplementary Figure 2: Comparison of three different optimizers available in PESTO on the provided `jakstat_signaling` example problem. Likelihood waterfall plots show sorted final log-likelihood values obtained from 25 optimization runs of each PSwarm, fmincon and MEIGO. Plateaus in the plot correspond to local optima, the plateau with the highest log-likelihood is assumed to represent the global optimum.
Total CPU time for the 25 starts were: PSwarm: 4361s, MEIGO: 3165s, fmincon: 1435s. The CPU time per start converged for to the global optimum were: PSwarm: *none converged*, MEIGO: 226s, fmincon: 239s. Matlab code with detailed settings for this case study are included in the PESTO repository.

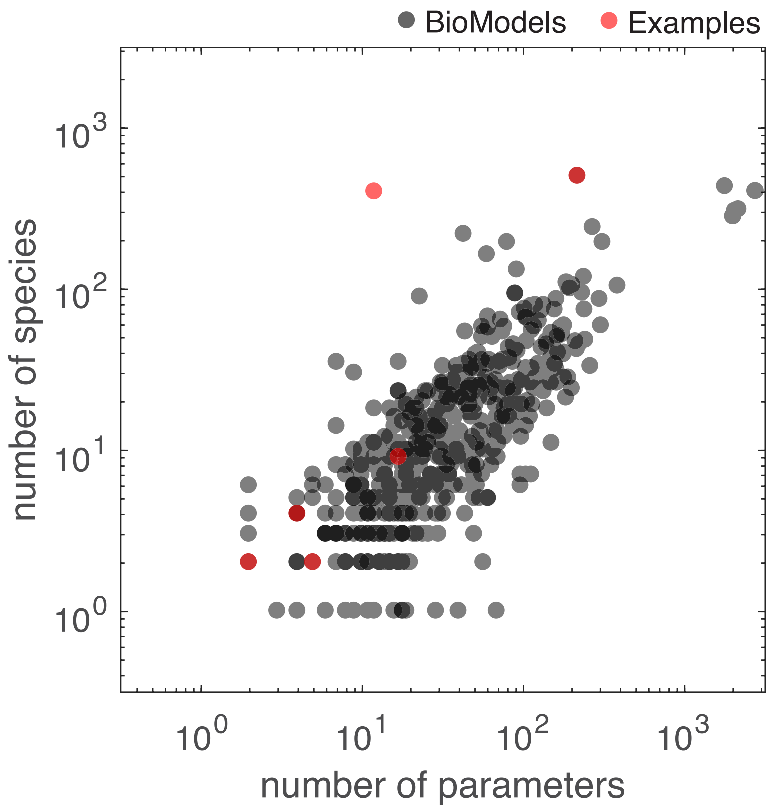

Supplementary Figure 3: Dimensionality of ODE models (in terms of number of species and parameters) contained in the BioModels database (`http://biomodels.net/`) (black points). Example models included in PESTO cover the size range of models used in current research (red points), demonstrating that PESTO can be applied to solve current parameter estimation problems in computational biology.
