# Supplementary material for: PESTO: Parameter EStimation TOolbox
Source: Bioinformatics. 2017 Oct 23;34(4):705–7. doi: 10.1093/bioinformatics/btx676 (PMC5860618; doi:10.1093/bioinformatics/btx676)
Supplement: Supplementary Data [file btx676_supp.zip › btx676-suppl_data/PESTO_Comparison.pdf]

Supplemental Table 1: Summary of software packages for parameter estimation as of October 2017, to the best of our knowledge.

		Supported Model types *				Parameter Optimization		Uncertainty Analysis										Visualization									
									Profile Likelihoods			Single Chain MCMC			Multi-Chain MCMC												
		ODE	PDE	SDE	Else	Multi-start local	Global Methods	Local Approximations	Optimization based	Integration based	Hybrid	Metropolis Hastings	Delayed Rejection Adapted Metropolis	Metropolis Adjusted Langevin Algorithm	Parallel Tempering	Parallel Hierarchical Sampling	Boot-strapping	Optimization	Local Approximations	Profile Likelihoods	MCMC Sampling	Boot-strapping	Confidence Intervals	Parallelization	Last update	Programming language	Website / Info
Modeling environments	AMIGO	Yes	Yes	Yes	Yes	Yes	Yes	Yes	No	No	No	No	No	No	No	No	Yes	Yes	Yes	No	Yes	Yes	Yes	No	2017	Matlab, Fortran	https://sites.google.com/site/amigo2toolbox/
	dmod	Yes	No	No	No	Yes	No	No	Yes	Yes	No	No	No	No	No	No	No	Yes		Yes	No	No	No	No	2017	R	https://cran.r-project.org/web/packages/dMod/
	d2d	Yes	No	No	No	Yes	Yes	Yes	Yes	No	No	No	Yes	No	No	Yes	No	Yes	Yes	Yes	Yes	No	Yes	pthreads, parfor	2017	Matlab	https://data2dynamics.github.io/d2d/
	Copasi	Yes	No	Yes	No	Yes	Yes	Yes	Yes	No	No	No	No	No	No	No	No	No	No	No	No	No	No	No	2017	standalone	http://copasi.org/
	PyCoTools	Yes	No	Yes	No	Yes	Yes	Yes	Yes	No	No	No	No	No	No	No	No	Yes	Yes	Yes	No	No	Yes	No	2017	Python, standalone	https://pypi.python.org/pypi/PyCoTools/2.0.2
	bioparkin	Yes	No	No	No	Yes	No	Yes	No	No	No	No	No	No	No	No	No	No	Yes	No	No	No	No	No	2015	Python, standalone	https://github.com/CSB-at-ZIB/BioPARKIN
	tellurium	Yes	No	No	No	No	No	No	No	No	No	No	No	No	No	No	No	No	No	No	No	No	No	No	2017	Python, C	http://tellurium.analogmachine.org/
Non-modeling environments	BioBayes	Yes	No	No	No	No	No	No	No	No	No	Yes	No	No	Yes	No	No	No	No	No	Yes	No	No	No	2010	Java, standalone	http://www.dcs.gla.ac.uk/BioBayes/
	CRAN: 'ProfileLikelihood'	No	No	No	Yes	No	No	No	Yes	No	No	No	No	No	No	No	No	No	No	Yes	No	No	No	No	2011	R	https://cran.r-project.org/web/packages/ProfileLikelihood/
	DOTcvpSB	Yes	No	No	No	Yes	Yes	No	No	No	No	No	No	No	No	No	No	Yes	No	No	No	No	No	No	2010	Matlab	http://nautilus.iim.csic.es/~dotcvpsb/
	DRAM	Yes	Yes	Yes	Yes	No	No	No	No	No	No	No	Yes	No	No	No	No	No	No	No	Yes	No	No	No	2006	Matlab	http://helios.fmi.fi/~lainema/dram/
	EVA 2	Yes	Yes	Yes	Yes	Yes	Yes	No	No	No	No	No	No	No	No	No	No	Yes	No	No	No	No	No	No	2015	Java, standalone	http://www.ra.cs.uni-tuebingen.de/software/eva2/features.html
	MEIGO	Yes	Yes	Yes	Yes	Yes	Yes	No	No	No	No	Yes	No	No	No	No	No	Yes	No	No	Yes	No	No	jPar	2014	Matlab, R, Python	http://gingproc.iim.csic.es/meigo.html
	**PESTO**	Yes	Yes	Yes	Yes	Yes	Yes	Yes	Yes	Yes	Yes	Yes	Yes	Yes	Yes	Yes	No	Yes	Yes	Yes	Yes	No	Yes	parfor	2017	Matlab	https://github.com/ICB-DCM/PESTO/
	sacess	Yes	Yes	Yes	Yes	Yes	Yes	No	No	No	No	No	No	No	No	No	No	No	No	No	No	No	No	MPI/OpenMP	2017	C, Fortran, Matlab	https://bitbucket.org/DavidPenas/sacess-library/overview
	SBML-PET-MPI	Yes	No	No	No	No	Yes	No	Yes	No	No	No	No	No	No	No	Yes	Yes	No	Yes	No	Yes	No	MPI	2011	C/C++/Fortran/Python/Matlab	https://sites.google.com/site/sbmlpetmpi/
	Sloppycell	Yes	No	No	No	Yes	No	Yes	No	No	No	No	No	No	No	No	No	Yes	Yes	No	No	No	No	MPI	2017	Python	http://sloppycell.sourceforge.net/

* ODE, PDE, SDE: ordinary, partial, stochastic differential equations
